# Variety Identification of Orchids Using Fourier Transform Infrared Spectroscopy Combined with Stacked Sparse Auto-Encoder

**DOI:** 10.3390/molecules24132506

**Published:** 2019-07-09

**Authors:** Yunfeng Chen, Yue Chen, Xuping Feng, Xufeng Yang, Jinnuo Zhang, Zhengjun Qiu, Yong He

**Affiliations:** 1 College of Biosystems Engineering and Food Science, Zhejiang University, Hangzhou 310058, China; 2 Institute of Horticulture, Zhejiang Academy of Agriculture Science, Hangzhou 310021, China

**Keywords:** orchids, variety identification, FTIR spectroscopy, stacked sparse auto-encoder

## Abstract

The feasibility of using the fourier transform infrared (FTIR) spectroscopic technique with a stacked sparse auto-encoder (SSAE) to identify orchid varieties was studied. Spectral data of 13 orchids varieties covering the spectral range of 4000–550 cm^−1^ were acquired to establish discriminant models and to select optimal spectral variables. K nearest neighbors (KNN), support vector machine (SVM), and SSAE models were built using full spectra. The SSAE model performed better than the KNN and SVM models and obtained a classification accuracy 99.4% in the calibration set and 97.9% in the prediction set. Then, three algorithms, principal component analysis loading (PCA-loading), competitive adaptive reweighted sampling (CARS), and stacked sparse auto-encoder guided backward (SSAE-GB), were used to select 39, 300, and 38 optimal wavenumbers, respectively. The KNN and SVM models were built based on optimal wavenumbers. Most of the optimal wavenumbers-based models performed slightly better than the all wavenumbers-based models. The performance of the SSAE-GB was better than the other two from the perspective of the accuracy of the discriminant models and the number of optimal wavenumbers. The results of this study showed that the FTIR spectroscopic technique combined with the SSAE algorithm could be adopted in the identification of the orchid varieties.

## 1. Introduction

Orchids, one of the two major families of flowering plants, have fascinated botanists and plant enthusiasts over centuries [1]. Since the introduction of tropical species into cultivation in the 19th century, a large number of orchid hybrids and cultivars have been produced by horticulturists. Many orchids are mistaken for other varieties because of the similar appearance of orchids. Thus, it is important to clarify the identity and type of an orchid and distinguish it from other similar orchid families. Normally, orchid taxa are determined by their main morphology, ecology, and rarity [2,3]. In addition, many experts determine the type of orchid by identifying the differences in genes [4,5], an expensive or time consuming process which is often applied to only a few sample seeds. Thus, it is of significant interest to develop a rapid method for identifying orchid varieties. In recent years, the spectroscopic technique has proven to be a powerful analytical tool that can provide detailed structural information on sample properties and composition at the molecular level [6]. Fourier transform infrared (FTIR) is considered a simple (requiring minimum sample preparation), rapid, low-cost, and high sensitivity applied spectroscopic method [7]. Like the fingerprints of each person, the infrared spectrum of any substance is known to be unique, which allows infrared spectroscopy to be applied to identify unknown samples or classify different samples [8]. FTIR spectroscopy has been applied in many classification studies, such as the discrimination of tea varieties [9], the classification of Moroccan olive cultivars [10], and a discrimination study between the grain of spelt and common wheat hybrids and their parental forms [11]. Thus, it is effective way to identify the type of orchids with similar appearance. Since different types of orchids contain diverse physiological information, orchids can be identified by the FTIR spectroscopy technique. FTIR spectroscopy produces a large amount of spectral information data which could increase analytical complexity and reduce computational efficiency. The multivariate analytical technique has a direct impact on performance after spectral data acquisition. Principal component analysis (PCA) [12], competitive adaptive reweighted sampling (CARS) [13], and the successive projections algorithm (SPA) [14] have been applied to reduce the dimensions of spectral data, and these methods have proven to be effective. However, these three methods are performed without supervision and only consider the internal relationship of the sample data variables, not including the label information to which the sample belongs. Some discriminant models with strong recognition have been used to handle with classification problems, such as the support vector machine (SVM) [15], k-nearest neighbor (KNN) [16], and partial least squares discriminant analysis (PLS-DA) [17]. The stacked sparse auto-encoder (SSAE) reconstructs the input variables into sparse feature representations under sparse constraints [18]. The whole process is end to end without supervision, and the SSAE learns the features of the input data during this process. After that, the decoding part of the SSAE is removed and one classifier is added at the end of the encoding part. Combining the SSAE with a classifier proved to improve its classification rate, especially when dealing with the large-scale data sets [19]. The combination of an SSAE and classifier has been applied to identify the nucleus on breast cancer histopathology images [18] and to detect striped stem borer infestation on rice hyperspectral data [20]. As FTIR data contains large-scale and high-dimensional information, this study used an SSAE with a softmax classifier to identify the type of orchid. In addition, through the reversing the derivation of the SSAE’s output, the optimal features could be selected from the original spectral data in this study, which was compared with other feature selection algorithms.

The purpose of this research was to explore three objectives: (1) To examine the feasibility of using FTIR spectroscopy to identify orchid leaves; (2) to compare the recognition results of the SSAE model with KNN and the SVM; and (3) to select the optimal wavenumbers that identify the differences among different orchid varieties.

## 2. Results and Discussion

### 2.1. Spectral Profiles

**Figure 1:**
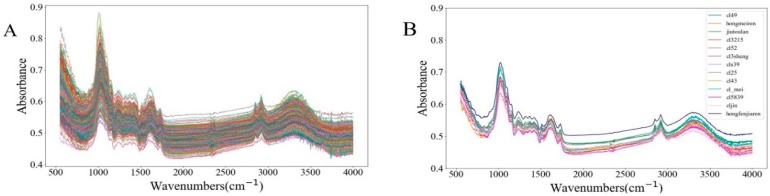
The profiles of (**A**) the raw spectra and (**B**) the average spectra of each orchid.

The spectra of all original data are shown in Figure 1A, and the average spectra of each species are shown in Figure 1B. Since each spectrum was obtained based on 32 scans, the spectral profiles are very smooth. For a more detailed analysis of the spectrum, a broad-band O–H stretch was observed near 3322 cm^−1^ (between 3200 and 3650 cm^−1^) [21]. The absorption peaks at 2849 and 2924 cm^−1^ are mainly attributed to the stretching vibration of C–H [22]. The peak at 1620 cm^−1^ is caused by the O–H bending of absorbed water [23]. The absorption peak at 1417 cm^−1^ is mainly caused by the combination of the C–H bending of the alkenes and the O–H bending of the C–OH group [24]. The peak at 1369 cm^−1^ mainly offers information about the C–H bending vibrations [25]. The absorption at 1237 cm^−1^ was the bending of O–H [26]. The largest absorption occurs near 1023 cm^−1^, mainly due to the C–O stretching in cellulose [27]. Obviously, there were differences in the spectral absorption rates of different orchid varieties, but we could not distinguish different types by spectral difference alone.

### 2.2. Discriminant Models Based on Full Spectra

Therefore, some chemometric methods were used to analyze the spectra and establish recognition models for different varieties. Firstly, the discriminant models were established by full spectra data. The KNN, SVM, and SSAE were used to establish the discriminant models, and the accuracy of the classification recognition was used as an evaluation metric of the model performance. As shown in Table 1, the classification accuracy of the SSAE model was 99.4% for the calibration set and 97.9% for the prediction set, both of which were significantly higher than those of the SVM and KNN models. It was clear that the performance of the KNN model was worse than the other two models—the accuracy for the prediction set was lower than 60%, and there was serious over-fitting. Though the calibration set accuracy rate of the SVM model reached 100%, the prediction set accuracy rate was 92.6%. There was a slight over-fitting for the SVM model. Therefore, the classification effect of the SSAE model was significantly better than the other two models. In the previous study, Nie et al. applied near-infrared hyperspectral imaging technology combined with a deep convolutional neural network to identify hybrid seeds and reached an accuracy of 93.87% and 96.12% for hybrid loofah and okra, respectively [28]. Wu et al. classified three varieties of tea samples using FTIR and the allied Gustafson–Kessel algorithm, a process which reached the accuracy of 93.9% at the wavenumber range of 4001.569–401.1211 cm^−1^ [9]. Michele et al. reported LDA (Linear Discriminant Analysis) models with discriminant accuracy of 92.0% at the wavenumber ranges of 2300–600 and 3000–2400 cm^−1^ for five different types of Moroccan olive cultivars [10]. Therefore, FTIR spectroscopy combined with the SSAE model for the identification thirteen different types of orchids performed better than previous works, both in terms of the number of experimental object varieties and the accuracy of recognition.

### 2.3. Feature Visualization with t-SNE

To visually demonstrate the validity of the dispersion of the SSAE network, the distribution of feature variables was visualized by projecting features from the high-dimensional space into the two-dimensional space based on t-distribution stochastic neighbor embedding (t-SNE). Then, the two-dimensional data were used to draw a scatter plot to observe the distribution of the raw data. Different from the traditional dimensionality reduction techniques that perform a linear mapping of data from a high to low dimensional space, t-SNE has been widely used to visualize high-dimensional data with a non-linear dimensionality reduction method.

**Figure 2:**
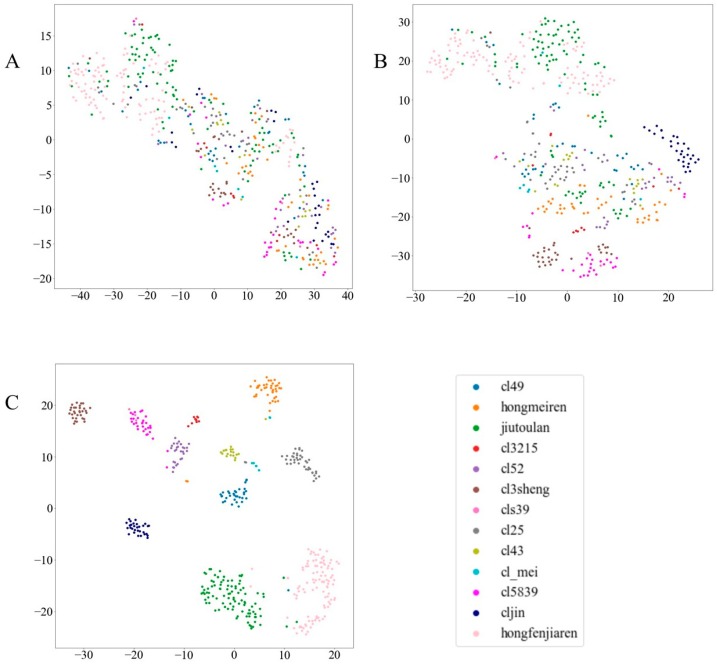
The visualization maps of (**A**) the raw spectral data, (**B**) the output of the stacked sparse auto-encoder (SSAE) hidden layer1 and (**C**) the output of the SSAE hidden layer2; the x–y axis of each figure corresponds to the two dimensions of the reconstructed space by t-distribution stochastic neighbor embedding (t-SNE).

As shown in Figure 2, t-SNE was adopted to visualize the original data and two-layer output features of the SSAE. The feature representation of hidden layer1 is more dispersed than the original spectral data. From the visualization of the original data, it can be seen that the distribution of different kinds of data is disorderly, and the feature representation of hidden layer1 had a certain degree of difference with respect to the raw data while being highly compact. In contrast, the feature representation of hidden layer2 was clearly separated, while individual samples were misclassified. This visualization technology had the potential to explain the effectiveness of the SSAE in processing the spectral data and to establish an intuitive visual differentiation method.

### 2.4. Optimal Wavenumber Selection

**Figure 3:**
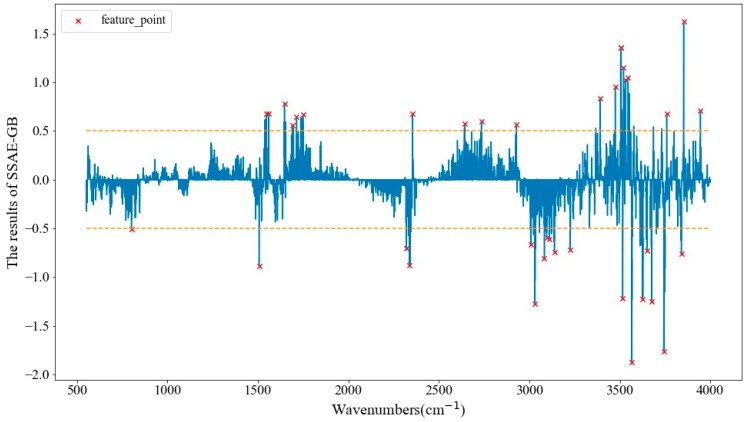
The partial guided result of stacked sparse auto-encoder guided backward (SSAE-GB) with selected wavenumbers.

**Figure 4:**
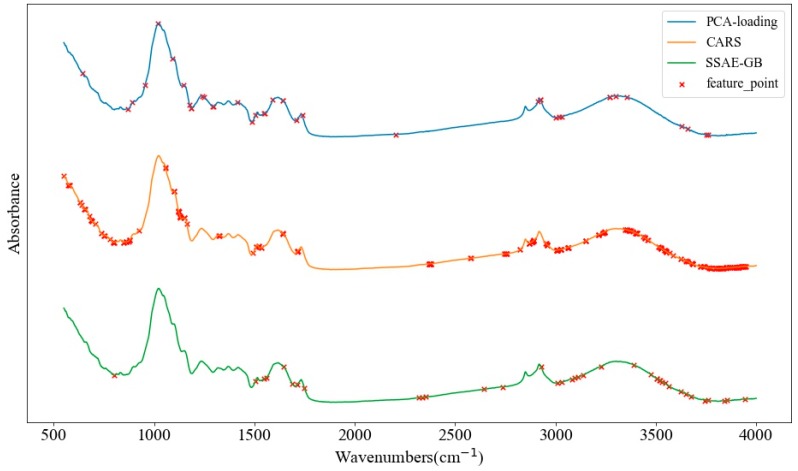
The distribution of optimal wavenumbers selected principal component analysis loading (PCA-loading), competitive adaptive reweighted sampling (CARS), and SSAE-GB.

For the development of instruments that can be adopted for portable detection, the use of characteristic spectroscopy can effectively reduce the development cost of the instruments, as processing the whole band of spectral data would increase the computational complexity and require more computing power of the hardware devices. Thus, the selection of sensitive wavenumbers in multivariate spectra analysis is necessary to determine principal spectral information and simplify the modeling process. PCA-loading, CARS and stacked sparse auto-encoder guided backward (SSAE-GB) were applied to select optimal wavenumbers in this study. As shown in Figure 3, the strong peaks and valleys of SSAE-GB’s partial guided result which had an absolute value over 0.5 were chosen as optimal wavenumbers, and 38 optimal wavenumbers were selected. The wavenumbers distribution of each selection method is displayed in Figure 4. PCA-loading selected 39 optimal wavenumbers, and CARS selected the most optimal wavenumbers to be 300, after which the number of wavenumber variables was significantly reduced by 95.81% and 99.47%, respectively. There were many overlapping ranges of the three methods. The maximum overlapping range was between 3000 cm^−1^ and 3750 cm^−1^, which can be assigned as O–H stretching [29]. The other common overlapping ranges included 1712 cm^−1^ (for C=O stretching [30]), 1642 cm^−1^ (for H–O–H bending of water [31]), 1530 cm^−1^–1560 cm^−1^ (for coupled NH deformation and C–N stretching [32]), and near 861 cm^−1^ (for CH2 rocking vibrations [33]).

To verify the validity of the feature wavenumbers selection, models of the SVM and KNN were established based on the optimal wavenumbers extracted by PCA-loading, CARS, and SSAE-GB. The recognition effect of each model for the calibration set and the prediction set is shown in Table 2. The SVM model performed much better than the KNN model, like the full spectrum modeling. In addition, most of the optimal wavenumbers-based models were slightly better than the full spectra models, which can be reflected in both the SVM model and the KNN model. This could indicate that the full spectrum with 7157 variables contained redundant information, which affected the accuracy of the model. Though the SVM model on optimal spectrum selected by CARS was slightly better than the SVM model based on the SSAE-GB method, the number of characteristic wavenumbers selected by SSAE-GB was 0.531% of the full spectrum, and the CARS method selected up to 4.192%. In contrast, SSAR-GB had satisfactory performance and was suitable for spectral feature selection.

## 3. Materials and Methods

### 3.1. Samples Preparation and FTIR Spectra Acquisition

**Figure 5:**
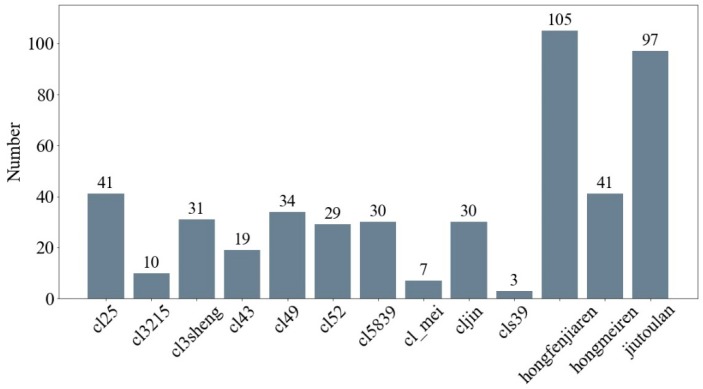
The number of each orchid.

Thirteen varieties of orchid leaves (including cl25, cl3215, cl3sheng, cl43, cl49, cl52, cl5839, cl_mei, cljin, cls39, hongfenjiaren, hongmeiren, and jiutoulan) were collected from the orchid nursery in the Institute of Horticulture, Zhejiang Academy of Agricultural Science, Hangzhou, China. The plants were collected from the same greenhouse to reduce environmental effects. The orchids whose names begin with ‘cl’ are hybrid combinations of Chunlan (Cymbidium goeringii) and other orchid species, and the orchids whose names begin ‘h’ or ‘j’ are of a different four-season orchid (Cymbidium ensifolium) variety. The number of experimental samples for each orchid is shown in Figure 5.

All samples were freeze-dried and grounded to a powder using a grinder before being subjected to FTIR spectroscopy over a wavenumber range of 4000–550 cm^−1^. Before each sample scan, 0.02 g of the sample was evenly mixed with 0.98 g of dried KBr powder, and the entire process was operated under an infrared heat lamp to minimize the variations in the moisture content of the sample powder. The uniformly mixed powder was pressed as 15 MPa for 30 s using a tableting machine. The FTIR spectra of these samples were obtained by using a FTIR spectrometer (FTIR 4100, JASCO Corporation, Tokyo, Japan) with a spectral resolution of 4 cm^−1^. During the spectral scanning, each sample was scanned 32 times, and the average spectra was taken as the sample spectra. The sampling interval of background signal was set to 45 min, and the entire experimental temperature was maintained at approximately 25 °C.

### 3.2. Multivariate Data Analysis

**Figure 6:**
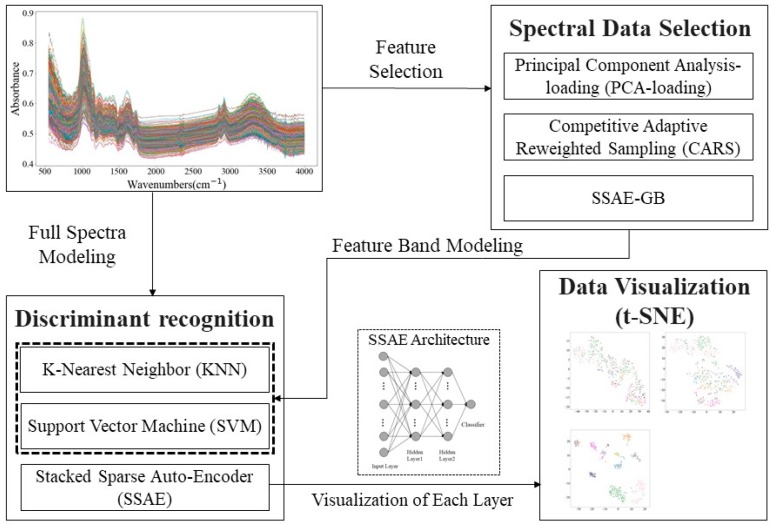
Experimental flow chart.

Three discriminant models, the KNN, SVM, and SSAE, were applied to identify the type of orchids based on the full band spectra. The KNN and SVM models were used to compare the representative ability of optimal wavenumbers selected by PCA-loading, CARS, and SSAE-GB. A high-dimensional dataset visualization method, t-SNE, was applied to explore the feature extraction ability of the SSAE. The multivariate data analysis was implemented in Python 3.6.8 and run by Jupyter 4.4.0. In addition, the flow chart of this research is shown in Figure 6.

#### 3.2.1. K-Nearest Neighbor

K-nearest neighbor (KNN) is a non-parametric method wildly used for pattern recognition, which is a non-parameters method [34]. It finds the k instances nearest to the unknown instance in the training dataset where k is a manually set parameter. The unknown instance is identified as the category to which majority of these k nearest instances belong. Determining the parameter k is critical for KNN. For this study, the optimal k was selected from 3 to 20 with a step of 1 by using three-fold cross validation.

#### 3.2.2. Support Vector Machine

The support vector machine (SVM) is a supervised recognition algorithm whose basic model is the linear classifier with the largest interval in the feature space [35]. Owing to the high efficiency of processing linear and non-linear data, the SVM has been widespread used in spectral data analysis. The SVM maps the data from the original space to the higher-dimensional feature space by the kernel function, such that instances of individual categories are separated as clearly as possible. For this study, the SVM with the radial basis function was applied. In order to make the SVM model achieve optimal performance, a grid-search procedure was used to obtain the optimal penalty parameters (c) and kernel function parameters (g) with the searching range of 2^−8^–2^8^.

#### 3.2.3. Principal Component Analysis loading

Principal component analysis (PCA) is an effective data dimension reduction method which has been widely applied in processing large spectral data [36]. It converts linearly correlated high-dimensional variables into linearly independent low-dimensional variables (called principal components) by orthogonal variation. The first principal component has the largest variance, and each succeeding component in turn has highest variance possible under the constraint that it is orthogonal to the preceding components. The main information of the raw variables is included in the first few principal components. PCA-loading is obtained based on the PCA algorithm, which can extract the characteristic variables by analyzing the loading. PCA-loading reflects the degree of correlation between the original spectral variables and the principal component. The larger the PCA-loading, the more important the band corresponding to the spectral variable is. In this study, the optimal spectra were found in the loading of the first 6 principal components which explained 98.65% of the total variance.

#### 3.2.4. Competitive Adaptive Reweighted Sampling

Competitive adaptive reweighted sampling (CARS) is a method for selecting feature bands by Monte Carlo sampling combined with the partial least squares regression model [37]. CARS combines an exponential decay function and the adaptive weighted sampling algorithm, selects variables by comparing regression coefficient weights of variables in the PLS model, and removes the variables with small weight. Besides, it selects the lowest RMSECV (Root Mean Squared Error Cross Validation) by interactive verification, which is an effective method to find the optimal combination of variables.

#### 3.2.5. Stacked Sparse Auto-Encoder

The stacked sparse auto-encoder (SSAE) is a multi-layered feature expression framework consisting of a layer-by-layer stack of sparse auto-encoder (SAE) structures which can be used to extract the key information from original data [38].

**Figure 7:**
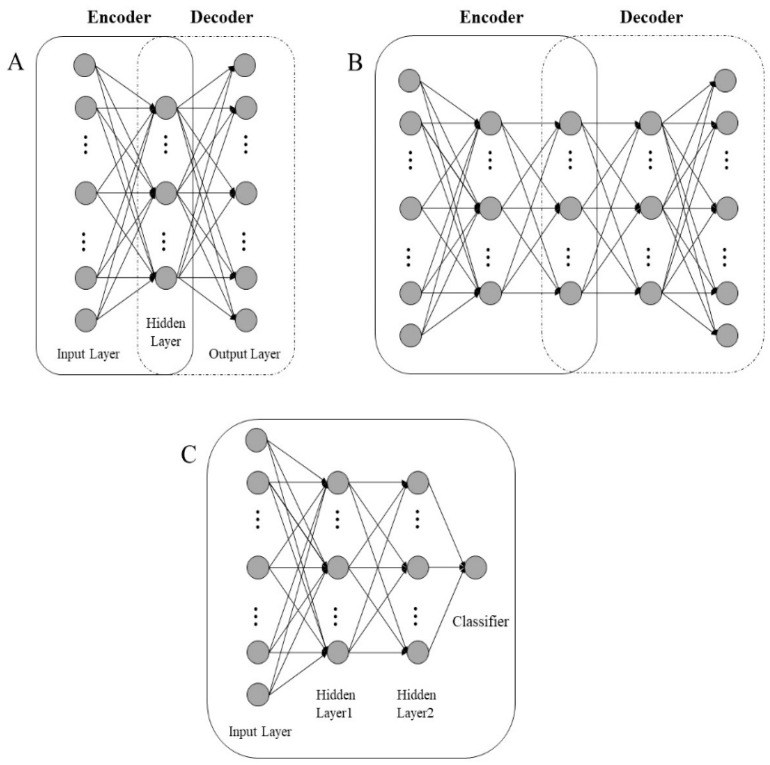
The structure of (**A**) basic sparse auto-encoder, (**B**) SSAE, and (**C**) SSAE with classifier.

A sparse auto-encoder (SAE) network shown is in Figure 7A. It is composed of one input layer, one hidden layer, and one output layer, which includes the encoder and decoder. The encoder and decoder are performed as follows:

(1) $$h=f\left(W_{1}x_{i}+\;b_{1}\right)$$

(2) $$z=f\left(W_{2}h+\;b_{2}\right)$$

where *h* is the feature representation; *W*_1_ and *b*_1_ are encoding weights and biases, respectively; relative *W*_2_ and *b*_2_ are decoding weights and biases; and *f*( ) is a sigmoid function ${\left(1+\exp\left(-x\right)\right)}^{-1}$, which is a nonlinear activation function introducing nonlinear characteristics for the feature representation process. The main purpose of the SAE is to reduce the dimensions of the input data by minimizing the error between input variables and output variables. Mathematically, it can be formulated as follows:

(3) $$L\left(X,\;Z\right)=\;\frac{1}{2}\sum_{i=1}^{m}||z_{i}-\;x_{i}{\left|\right|}_{2}^{2}+\;\beta \sum_{j=1}^{n}KL(\rho \;||\;{\hat{\rho }}_{j})$$

where *x* and *z* are the input and reconstructed output, respectively; *m* is the number of training dataset; and *n* is the number of features of *W*. Here, $KL(\rho ||{\hat{\rho }}_{j})$ is the Kullback–Leibler (KL) divergence [39]. The KL divergence is adopted to penalize the difference between ρ and ${\hat{\rho }}_{j}$ so that only a few hidden units respond to specific categories and most are suppressed, denoted as follows:

(4) $$KL(\rho ||\hat{\rho })=\;\rho \log\frac{\rho }{{\hat{\rho }}_{j}}+\left(1-\;\rho \right)\log\frac{1-\;\rho }{1-\;{\hat{\rho }}_{j}}$$

where ρ is a sparse parameter that is usually set to a small value close to 0 and ${\hat{\rho }}_{j}$ is the average activation value of the hidden unit *j* on all data in the training set.

As shown in Figure 7B, multiple SAEs build a SSAE network in which the characteristic representation *h* of each SAE is the input of the continuous SAE.

In this study, the encoder of the SSAE was pre-trained, and the decoder was removed, with the last coding unit followed by a classifier (Figure 7C). The softmax loss function was used as a classifier to fine-tune the entire SSAE network in a supervised manner, which can indicate the ability of the model to fit the tagged data. The activation function of the softmax classifier, $h_{\theta }\left(x\right)$, determines the probability P(y=j |x; θ) that x belongs to the category j. $h_{\theta }\left(x\right)$ was defined as follows:

(5) $$h_{\theta }\left(x_{i}\right)=\;\left[\begin{array}{c} \begin{array}{c} P(y_{i}=1\;|x_{i};\;\theta )\\ P(y_{i}=2\;|x_{i};\;\theta ) \end{array}\\ \vdots \\ P(y_{i}=k\;|x_{i};\;\theta ) \end{array}\right]=\;\frac{1}{\sum_{j=\;1}^{k}e^{\theta_{j}^{T}x_{i}}}\left[\begin{array}{c} \begin{array}{c} e^{\theta_{1}^{T}x_{1}}\\ e^{\theta_{2}^{T}x_{2}} \end{array}\\ \vdots \\ e^{\theta_{k}^{T}x_{k}} \end{array}\right]$$

where k is the number of SSAE’s last coding layer output and $\theta_{j}$ is the parameter that maps x to the *j*th category, which was specified by the classifier. In addition, the loss function of the entire supervised model including the coding layers of the SSAE and the classifier is defined as follows:

(6) $$J\left(\theta \right)=\;-\frac{1}{m}\left[\sum_{i=1}^{m}\sum_{j=1}^{k}1\left\{y_{i}=j\right\}\log\left(P(y_{i}=j\;|x_{i};\;\theta )\;\right)\right]+\;\frac{\lambda }{2}{\left||\theta |\right|}_{2}$$

where λ is weight decay coefficient which can prevent overfitting and ${\left||\theta |\right|}_{2}$ is L2 norm of the parameters in $h_{\theta }\left(x\right)$. $1\left\{y_{i}=j\right\}$ is the indicator function, defined as follows:

(7) $$1\left\{y_{i}=j\right\}=\;\{\begin{array}{c} 1\;if\;y_{i}=j\\ 0\;if\;y_{i}\;\neq j \end{array}$$

It can be seen from $1\left\{y_{i}=j\right\}$ that if sample $x_{i}$ of class j is misclassified, the $P(y_{i}=j\;|x_{i};\;\theta )$ is very small, and the corresponding J(θ) would be large because of the logarithmic mapping. Thus, with backward learning, the learning trend of θ is to make $P(y_{i}=j\;|x_{i};\;\theta )$ bigger in order to implement the process of optimizing parameters.

It is well known that the SSAE has some hidden layers, and it is hard to choose characteristic variables only based on the weights of the first network layer. In order to consider the features learned by each layer, this study we used the method which takes a reverse derivative of J(θ) called the stacked sparse auto-encoder guided backward (SSAE-GB). As described in the following equation, we found the partial guide of $x_{i}$ for J(θ) which could be used to select the optimal variables. As shown by Equations (8) and (9), the larger absolute value of SSAE-GB’s partial guided result, the more important the absorbance at the wavenumbers corresponding to these values.

(8) $$\frac{\partial \;J\left(\theta \right)}{\partial \;P(y_{i}=j\;|x_{i};\;\theta )}=\;-\frac{1}{m}\sum_{j=1}^{k}1\left\{y_{i}=j\right\}\;\frac{1}{P(y_{i}=j\;|x_{i};\;\theta )}\;P^{\prime }(y_{i}=j\;|x_{i};\;\theta )$$

(9) $$\frac{\partial \;P(y_{i}=j\;|x_{i};\;\theta )}{\partial \;x_{i}}=\frac{\sum_{l=1}^{k}\left[\left(\theta_{j}^{T}-\;\theta_{l}^{T}\right)e^{\left(\theta_{j}^{T}-\;\theta_{l}^{T}\right)x_{i}}\right]}{{\left(\sum_{l=1}^{k}e^{\;\theta_{l}^{T}x_{i}}\right)}^{2}}$$

## 4. Conclusions

The results of this study indicated that 13 orchids genotypes could be identified by FTIR spectroscopy combined with appropriate multivariate analysis techniques. Three discriminant models, the SSAE, SVM, and KNN, were built on the full spectra of 4000–550 cm^−1^ obtained by the FTIR spectroscopy technique. Each model was operated in three-fold cross validations; the SSAE model performed more effectively than the SVM and KNN based on results of the calibration set and prediction set. The SSAE model achieved the classification accuracy rates of 99.4% and 97.9% for the calibration set and prediction set, respectively. Though the SVM model’s accuracy of the calibration set reached an amazing 100%, the accuracy of the prediction set only reached 92.6%, and there was a certain over-fitting phenomenon. In order to further observe the SSAE model from another aspect, the feature distribution of each layer was visualized by t-SNE, and the visualized results demonstrated that it was feasible to identify the type of orchids using the SSAE model. At the same time, three feature selection methods, PCA-loading, CARS, and SSAE-loading, were used to reduce the dimensions of the data variable, which selected 39, 300, and 38 characteristic wavenumbers, respectively. The SVM and KNN models built on the optimal wavenumbers were used to compare the effects of the three feature selection methods. The accuracy of the SVM was much better than the KNN model, having the same phenomenon as these two models built on the full band. The prediction set’s accuracy of the SVM model based on the characteristic wavenumbers selected by SSAE-GB was 94.5%, which was slightly lower than the accuracy of the SVM based on the CARS method. However, the number of characteristic features selected by SSAE-GB was 38—extremely less than the 300 features of the CARS method. Comprehensively, SSAE-GB performed more effectively than the other two. This analytical protocol undoubtedly has great prospects in orchid identification, as it provides a quick and effective detection method. Future experiments will enrich the spectral database of orchids to improve the robustness and generalization of the recognition model so as to identify orchids more quickly and efficiently.

## Figures and Tables

**Table 1:** Classification results of discriminant models based on full spectra.

Model	Parameter ^a^		Accuracy (%)							
			Calibration Set				Prediction Set			
			1	2	3	Mean	1	2	3	Mean
**KNN**	K	3	78.0	81.1	81.1	80.1	61.6	60.4	47.8	56.6
**SVM**	(c, g)	(256, 0.035)	100.0	100.0	100.0	100.0	94.3	92.4	91.2	92.6
**SSAE**	(h1, h2)	(2048, 13)	99.1	99.7	99.4	99.4	98.7	98.1	96.9	97.9

1, 2, 3: the results of the three-fold cross validation; ^a^ K: The number of nearest neighbors; c: The penalty parameter of the SVM model; g: The parameter of kernel function; h1: The number of the hidden layer1 neuron nodes of the SSAE model; h2: The number of the hidden layer2 neuron nodes of the SSAE model.

**Table 2:** Detection accuracy of multiple characteristic wavenumbers methods.

	SVM			KNN		
	Parameters ^a^ (c, g)	Mean Accuracy (%)		Parameters ^a^ (K)	Mean Accuracy (%)	
		Calibration	Prediction		Calibration	Prediction
**PCA-loading**	(256, 4)	99.2	90.1	3	77.3	57.4
**CARS**	(256, 0.5)	99.9	95.2	3	80.1	61.4
**SSAE-GB**	(256, 4)	99.7	94.5	3	84.3	68.5

^a^ c: The penalty parameter of the SVM model; g: The parameter of kernel function; K: The number of nearest neighbors.
